# Defects in Electron Beam Melted Ti-6Al-4V: Fatigue Life Prediction Using Experimental Data and Extreme Value Statistics

**DOI:** 10.3390/ma14030640

**Published:** 2021-01-30

**Authors:** Viktor Sandell, Thomas Hansson, Sushovan Roychowdhury, Tomas Månsson, Mats Delin, Pia Åkerfeldt, Marta-Lena Antti

**Affiliations:** 1 Division of Materials Science, Luleå University of Technology, 731 87 Luleå, Sweden; pia.akerfeldt@ltu.se (P.Å.); marta-lena.antti@ltu.se (M.-L.A.); 2 Division 9654: Materials Engineering, GKN Aerospace, 461 38 Trollhättan, Sweden; thomas.no.hansson@gknaerospace.com; 3 Division 9641: Solid Mechanics, GKN Aerospace, 461 38 Trollhättan, Sweden; sushovan.roychowdhury@gknaerospace.com (S.R.); tomas.mansson@gknaerospace.com (T.M.); 4 Division 9633: Materials and Inspection Technologies, GKN Aerospace, 461 38 Trollhättan, Sweden; mats.delin@gknaerospace.com

**Keywords:** additive manufacturing, electron beam melting, Ti-6Al-4V, defects, fatigue life, fracture mechanics, fatigue crack propagation, probabilistic modeling

## Abstract

Electron beam melting is a powder bed fusion (PBF) additive manufacturing (AM) method for metals offering opportunities for the reduction of material waste and freedom of design, but unfortunately also suffering from material defects from production. The stochastic nature of defect formation leads to a scatter in the fatigue performance of the material, preventing wider use of this production method for fatigue critical components. In this work, fatigue test data from electron beam melted Ti-6Al-4V specimens machined from as-built material are compared to deterministic fatigue crack growth calculations and probabilistically modeled fatigue life. X-ray computed tomography (XCT) data evaluated using extreme value statistics are used as the model input. Results show that the probabilistic model is able to provide a good conservative life estimate, as well as accurate predictive scatter bands. It is also shown that the use of XCT-data as the model input is feasible, requiring little investigated material volume for model calibration.

## 1. Introduction

Additive manufacturing (AM) of metals using powders as the feedstock material is developing to become a viable process for the aerospace industry [1]. One of the main challenges with powder-based AM techniques is achieving a defect-free material to optimize the fatigue life [2]. The primary origin for crack initiation in powder bed AM materials is the rough, as-produced, surface [3,4]. For a material for which the surface is treated or removed in order to smoothen it, volumetric defects such as a lack of fusion, gas porosity, keyhole porosity, or inclusions are the primary sources for crack initiation [4,5,6]. In 2016, Seifi et al. [7] highlighted the need to account for AM defect populations in fatigue life modeling. Fracture mechanical models (Kitagawa–Takahashi-type diagrams) have been shown to yield good results for fatigue strength predictions [8,9,10,11,12]. For deterministic modeling of fatigue life, fatigue crack growth (FCG) methods have been used [6,12,13,14]. These models are based on defect size measurements. Examples of different FCG-based models include the use of different crack growth descriptions such as the Paris–Erdogan equation [15] or variations of the NASGRO equation [3,14,16].

Seifi et al. [7] also advocated for the use of probabilistic modeling of fatigue behavior, i.e., models taking statistical variance into account in contrast to the deterministic models. A common probabilistic approach is to use deterministic FCG models fed probabilistically derived input parameters [17]. Probabilistic models are useful considering the numerous variables introducing scatter in the fatigue behavior of AM materials [7,18].

As demonstrated in the 1980s in the works of Murakami [19], defects close to the surface are more likely to initiate crack growth than defects further from the surface. This has also more recently been shown to be the case for defects in AM materials [20,21,22]. Due to the generally large number of defects present in AM materials, there is almost always a near-surface defect determining the fatigue life [23]. In general, when defects are present, the fatigue failure of metal is determined by the largest defect experiencing the highest stress [24]. Thus, probabilistic models for AM need to, at a minimum, include the variability in the size and position of defects. This was done by Beretta, Romano et al. in a series of studies on a additively manufactured AlSi10Mg. They evaluated the tails of the defect size distributions with extreme value statistics and assumed random locations of defects. Both the position and size were measured mainly with non-destructive X-ray computed tomography, but also with classic metallography, and the results showed the accurate prediction of fatigue strength [14,25,26]. For a broader overview of the effects of defects on fatigue performance, including different modeling approaches, readers are referred to the review article by Sanaei and Fatemi [27].

Defects in AM materials are of different types having different origins in the building process [27]. Distinct defect types show differences in both size and shape, factors that influence crack initiation behavior. Lack of fusion (LOF) defects are a result of under-melting during processing [28,29,30], and keyhole porosity is a result of over-melting and partially vaporizing the metal during production [30,31]. Another defect type is gas pores coming from the powder or the building process. The two former defect types can be avoided by correct processing parameters, but gas pores are generally considered unavoidable [32].

Investigations of defects and their distribution have traditionally been carried out on 2D (or composite 3D) images using metallography [33], with X-ray computed tomography as an increasingly viable option. Recent advances in XCT have facilitated the documentation and analysis of complete 3D defect distributions even though there are limitations, primarily in regards to resolution, due to which certain types and sizes of defects are more difficult to reliably detect than others [23]. XCT also becomes less effective for high-density materials due to increased X-ray attenuation [34]. Compared to metallography, XCT is more costly to run, but compensates for that by being less time consuming and more efficient for larger material volumes [26]. Increased use of the XCT technique has also given rise to a shift in the method of choice for statistical analysis of life-determining defects. When relying on metallographic characterization, so-called block maxima (BM) statistical sampling is used [35]. In this method, the investigated material is subdivided into blocks, and the largest defect in each block is recorded. The data can then be described using a Gumbel distribution [36]. For XCT derived data, the use of peak-over-threshold (POT) has been shown to, with the same accuracy, be both quicker and simpler than BM sampling. POT sampling consists of recording all data points followed by truncation of the data at a threshold *u*. Such truncated data can be described using a generalized Pareto distribution (GPD) [37].

The present work aims to predict fatigue life in electron beam melted Ti-6Al-4V using different sets of known information regarding the defect population. One of the ways this is done is using a probabilistic approach based on X-ray computed tomography characterization. Extreme value statistical analysis of the distribution of internal defects is used to simulate the input for fatigue crack growth calculations. Validation is performed by comparison with empirical fatigue test data.

## 2. Materials and Methods

The material investigated was Ti-6Al-4V produced by electron beam melting (EBM) in an Arcam Q20plus machine. The powder used for the production was pre-alloyed Ti-6Al-4V extra low interstitial (ELI) powder from AP&C Inc. (Boisbriand, Québec, Canada), conforming with ASTM F3001 [38]. The chemical composition of the powder was in good agreement with ASTM F3001, as can be seen in Table 1.

**Figure 1:**
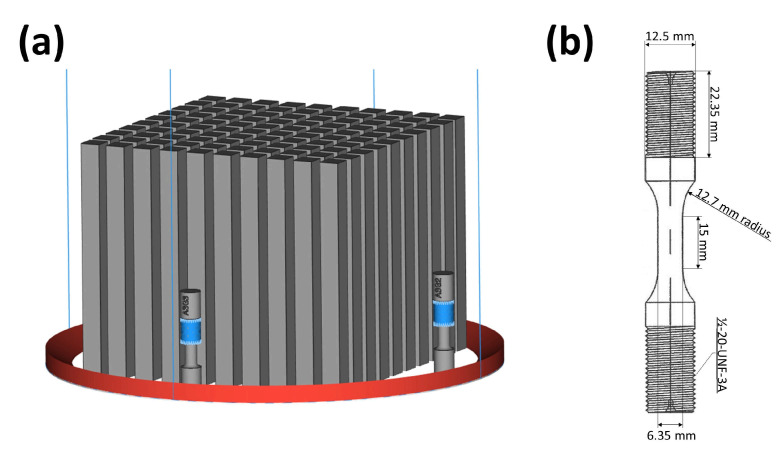
(**a**) Build layout in the Q20 machine’s cylindrical build chamber. The smaller objects in the build chamber are tensile test specimens for quality control. (**b**) Fatigue specimen geometry.

Two separate builds, named A and B, were produced with different build layer heights. Build A had a 50 μm layer height, and Build B had a 90 μm layer height. A rotating hatch was used with 67.5 degrees of rotation between layers. Standard EBM pre-heating scanning between layers kept the build at elevated temperature during production. The build layout is shown in Figure 1a. Arcam machines operate at a set voltage to power input during hatching that was equal in both builds. To get the required greater melt pool depth in Build B, the speed function was adjusted from 35 to 60 (procedure defined units) so that the scans were slower, increasing the energy density input. The building rate was ∼0.11 kg/h for Build A and ∼0.15 kg/h for Build B.

Four vertically built bars with dimensions of 15 mm × 15 mm × 180 mm were used from each build, A and B, for this study. From each of these bars, two fatigue specimens were machined for fatigue testing. In total, sixteen specimens were manufactured. Apart from blasting for powder cake removal, no further post-production processing was done. All specimens were machined so that the fatigue loading direction was parallel to the building direction. Each fatigue specimen was denoted AL, AH, BL, or BH, with the first letter signifying the build and the second letter indicating if the specimen came from the higher (H) or lower (L) half of the bar. The fatigue specimen dimensions are shown in Figure 1b.

**Figure 2:**
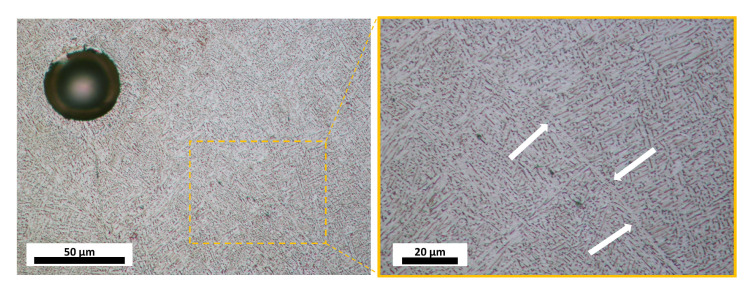
Typical microstructure in the observed material. A medium sized pore is visible. White arrows highlights an example of grain boundary α. Images are taken in the plane perpendicular to the building direction.

Etching with Kroll’s reagent revealed a typical prior-β structure elongated in the building direction with a microstructure predominately made up of fine basket weave α-laths with visible grain boundary α at prior-β grain boundaries, as can be seen in Figure 2.

Fatigue testing was carried out in an MTS 810 load frame equipped with a closed-loop servo-hydraulic system managed by an MTS TestStar IIs control system. The fatigue specimens were divided into two sets. Twelve specimens were tested under strain control and four under load control. The strain controlled testing used 1% strain with an R-ratio of −1 and a testing frequency of 0.5 Hz. The load controlled testing used 500 MPa with an R-ratio of 0 and a testing frequency of 10 Hz. All tests were run with sinusoidal loading until failure. Fractography was carried out using a JEOL JSM-IT300LV scanning electron microscope.

X-ray computed tomography (XCT) was carried out on the gauge lengths of the twelve fatigue specimens tested under strain control using a Zeiss Xradia 510 versa microtomograph from Carl Zeiss X-ray Microscopy (Pleasanton, CA, USA) employing a cone-beam setup. The parameters for the scans are presented in Table 2. Segmentation and analysis of the tomography data were carried out using Dragonfly PRO 4.1 [39] employing a segmentation algorithm for internal porosity specified in [20]. Any segmented volume smaller than 81 voxels was considered unreliable data and therefore excluded from the analysis. The reason for the 81 voxel limit is that the effective resolution was assumed to be 27 voxels, as is the case for a perfect scan [34]. Since one of the segmentation steps was applying a 3 × 3 × 3 median filter for denoising, the effective resolution consequently became 3 times larger than 27 voxels. For measurements of the defect size, equivalent diameters were calculated from the area of each defect projected on the plane perpendicular to the building direction.

### 2.1. Statistical Simulations of Defect Size Distributions

**Figure 3:**
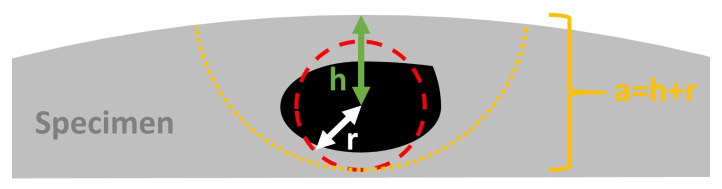
Definitions of the equivalent radius (*r*) and location (*h*) of a defect according to the Murakami criterion (1) [24]. The depth parameter *a* used for calculations was evaluated as a=h+r.

Size, shape, and location are the main factors determining a defect’s impact on fatigue. The largest defect, with the sharpest edges, at the location submitted to the highest stress shows the highest stress intensity factor and thus governs the fatigue behavior of a component [24]. For the porosity distribution simulations two assumptions were made: (i) Defect projections in the plane perpendicular to the loading direction were assumed to be circular. This is a good, but not perfect assumption based on the observed XCT data [20]. (ii) The defect location distribution was assumed to be uniform in the plane perpendicular to the building direction. Defect distributions (size+position) were simulated for one specimen volume, and individual defects were considered as near-surface if they fulfilled the criterion proposed by Murakami [24],

(1) r/h>0.8

where *r* is the equivalent radius of a circle with the same area as the defect and *h* is the distance from the center of the defect to the specimen surface, as shown in Figure 3. Only near-surface defects were used for crack growth calculations.

Defect size was defined as an equivalent defect diameter, *d = 2r*, calculated from the area of projection along the loading direction. The defect size distribution was approximated by a generalized Pareto distribution (GPD) according to the peak-over-threshold (POT) extreme value statistics method in which only the tail of the distribution above a certain cut-off diameter *u* is analyzed [37]. The tails of the defect size distribution as evaluated by XCT have previously been found to be exponentially distributed [20]; consequently, the shape parameter ξ of the GPD approaches 0, and the cumulative density function of the generalized Pareto distribution becomes a two-parameter exponential distribution:

(2) $$\begin{array}{cc} F_{GPD}\left(x,u,\sigma ,\xi \right)=F_{exp}\left(x,u,\sigma \right)=1-exp\left(-\frac{x-u}{\sigma }\right) & \;\mathrm{if}\;\xi \to 0 \end{array}$$

where σ is a scale parameter for the distribution of defect sizes larger than the cut-off parameter *u*. The *p*th percentile of the distribution can be derived from Equation (2) as:

(3) $$\begin{array}{c} x(p)=u-\sigma \cdot log(1-p) \end{array}$$

where *p* is the cumulative probability of recurrence. For each value of a distribution, the cumulative probability of recurrence can be associated to a return period *T* as p=1−1/T. The return period *T* of a maximum defect size in a characteristic volume *V* is:

(4) $$\begin{array}{c} T=\frac{N_{u}}{V_{0}}V=\rho V \end{array}$$

where $N_{u}$ is the number of observed defects larger than the cut-off value *u* in $V_{0}$, where $V_{0}$ is the investigated volume. Thus, ρ is the observed numerical density of defects larger than *u* in $V_{0}$. The characteristic maximum defect size ($\hat{x}\left(T\right)$) is defined as the size that, on average, is expected to be exceeded by at least one defect in a characteristic volume *V*, which can be expressed as:

(5) $$\begin{array}{c} \hat{x}\left(T\right)=\hat{u}+\hat{\sigma }\cdot log\left(T\right) \end{array}$$

where $\hat{u}$ and $\hat{\sigma }$ are the values of the cut-off and scale estimated from the XCT data. The cut-off parameter was evaluated graphically by fitting exponential percentile plots to truncated data in which exceedances formed a straight line whilst making sure not to select a cut-off such that the true initiator diameters as measured by fractography were excluded. The scale parameters was then evaluated using the maximum likelihood procedure. The distributions of a depth parameter (*a*), which is a new stochastic variable, were taken as:

(6) a=h+r

as illustrated in Figure 3, where the defect size radius *r* is taken as half of the defect diameter *d* randomly sampled from the defect size distribution described by the parameters $\hat{u}$ and $\hat{\sigma }$. The distance from the surface was sampled by assuming a homogeneous distribution in the cylindrical specimen volume, thus randomizing the distance from the specimen surface as:

(7) $$\begin{array}{c} h=D\sqrt{U(0,1)} \end{array}$$

where *D* is the test specimen diameter and *U* is the uniform distribution. As the tested and simulated volumes were equal and the number of defects in each volume far from zero, the number of simulated defects for each specimen was taken to be $∼N(\mu^{*},\sigma^{*})$ where $\mu^{*}$ is the empirical mean number of defects and $\sigma^{*}$ is the empirical standard deviation of the number of defects, in one specimen. For each specimen, the largest a=h+r fulfilling the Murakami rule (1) was recorded. This sampling was done 5000 times for each build as this was considered a sufficient number of data points to limit the variability of the evaluated parameter and small enough to give a reasonable calculation time.

### 2.2. Fatigue Crack Growth Calculations

**Figure 4:**
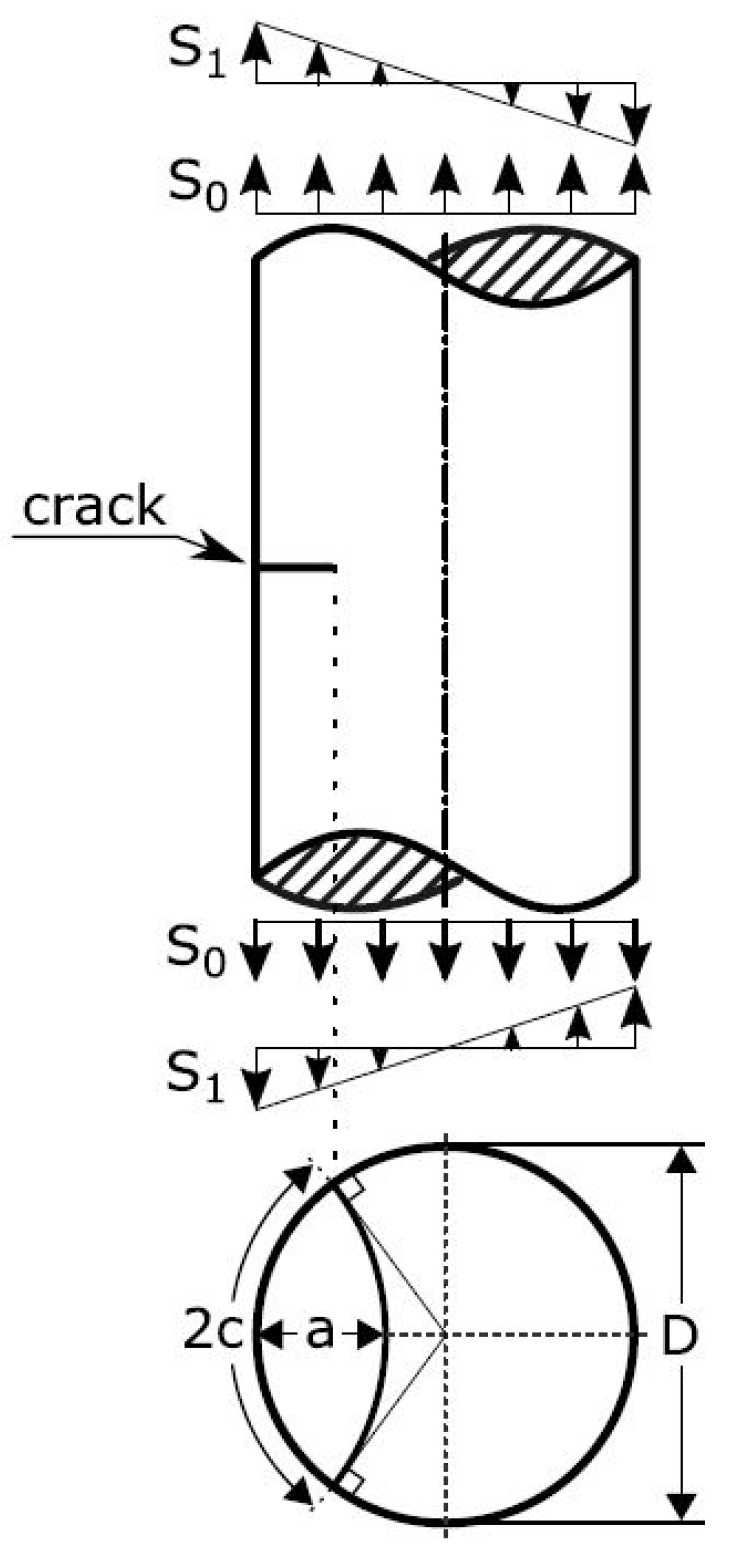
SC07-“semi-elliptical surface crack in solid cylinder”- crack case configuration [40]. Either *a*, the crack depth, or *c*, the circumferential crack length, can be used as the geometrical input parameter. S0 is uniform stress and S1 bending stress.

**Figure 5:**
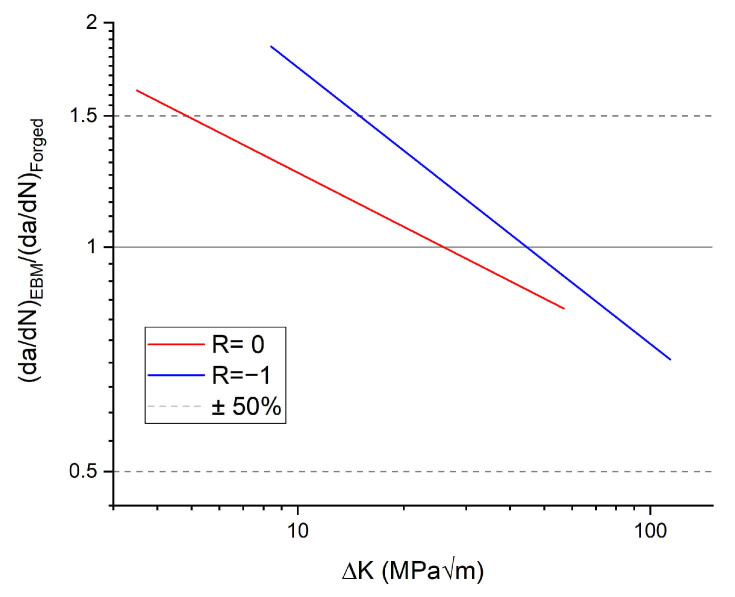
Comparison between the Paris descriptions of crack propagation data from electron beam melting (EBM) produced and forged Ti-6Al-4V for two stress ratios, R=0 and R=−1.

The commercial software NASGRO v9.10 [40] was used to simulate the fatigue life of the test specimens based on fractography and XCT results, as well as probabilistic modelling. NASGRO uses a library of two-dimensional crack cases for the calculation of the stress intensity factor. The crack case used in this work, “SC07 - semi-elliptical surface crack in solid cylinder”, is shown in Figure 4. The stress intensity factor range of the simulated defects was compared to an NASGRO equation description of forged Ti-6Al-4V crack propagation data from the GKN Aerospace company database. This showed only a slight overlap with the near-threshold regime of the crack growth curve. This overlap occurred at crack lengths similar in scale to the intrinsic crack length $a_{0}$ defined in the material description of the forged material. $a_{0}$ is the crack size at which the fatigue limit stress level gives rise to a stress intensity factor range equaling the threshold stress intensity factor range ($\Delta K=\Delta K_{th}$). This similarity in scale between the considered cracks and $a_{0}$ leads to an approximate decrease of $\Delta K_{th}$ by 30% due to the small crack effect [40]. The effect on the life of the accelerating crack propagation as $\Delta K\to K_{c}$ was considered negligible due to the limited number of cycles in this crack growth regime. On the basis of these two observation, Paris’ law descriptions of the crack propagation at R=0 and R=−1 were considered appropriate for the life analysis. Proprietary data from crack propagation tests of the tested EBM material at R=0 and R=−1 were then fit to Paris’ law and used for the crack propagation calculations. A comparison of a Paris description of the forged data to the EBM data is shown in Figure 5. At no stress, the intensity factor range was the difference in crack propagation rate larger than a factor of two. Modeling using forged data would thus provide less conservative predictions since most of the crack growth takes place at low ΔK levels.

As the input parameter in NASGRO, the depth parameter *a* was used. *a* was defined as the distance from the furthest edge of the initiating defect to the specimen surface (a=h+r in Figure 3). Only uniform stress was considered. The loads used for the FCG calculations of the strain controlled R=−1 specimen were taken from the empirical mid-life cycle loads. A comparison with calculations using true loads was made with negligible difference in predicted life. For failure evaluation, the net-section stress failure criterion in NASGRO was disabled. Instead, the calculations ended when the calculated maximum stress intensity factor exceeded the fracture toughness ($K_{c}$) set to 1799 $MPa\sqrt{mm}$, the same as the forged reference material. A similar fracture toughness has been reported for as-built EBM produced Ti-6Al-4V by Galarraga et al. [41]. Seifi et al. [18] reported a higher fracture toughness, but also a large variation within single builds, motivating the use of the more conservative value.

### 2.3. Fatigue Life Predictions

Fatigue life predictions using fatigue crack growth calculations were made in three ways. The first way was deterministic prediction from fractography, where the FCG input parameter *a* was measured from the fracture initiating defects found on fracture surfaces. The second way was deterministic prediction from XCT data using the deepest (largest *a*) observed defect fulfilling the Murakami criterion in each fatigue specimen. The third way was probabilistic prediction from XCT based on the distributions of *a* evaluated from the simulations of the specimen as described in Section 2.1. By running FCG calculations for a range of loads at R=0 and R=−1, prediction curves were created. The input for the calculations of the prediction bands were the 2.5th and 97.5th percentiles of the *a* parameter distributions. To complement the probabilistic model, a conservative fatigue life prediction curve was included. It was calculated similarly to the prediction band, but as the input for FCG calculations, the characteristic maximum defect size (diameter), $\hat{x}{\left(T\right)}_{1000}$, in a characteristic volume $V_{1000}$, as described in Equation (5), was used. $V_{1000}$ was taken to be the volume of 1000 hollow cylinders (specimen surfaces) with a width corresponding to the greatest sampled depth parameter (*a*) and a length equal to that of the specimen gauge length. Putting $a=\hat{x}\left(T\right)$ is the same as assuming that the edge of the characteristic maximum defect is tangential to the specimen surface.

## 3. Results

### 3.1. Fatigue Testing and Fractography

Table 3 shows the fatigue testing results for all specimens and the corresponding maximum and minimum stresses. The table also shows the defect size measurements from fractography, together with the associated predicted fatigue life values. Specimens with names ending with a number 1 to 3 were tested under strain control (R=−1 and 1% strain), and specimens with a 4 were tested under stress control (R=0 and 500 MPa stress).

**Figure 6:**
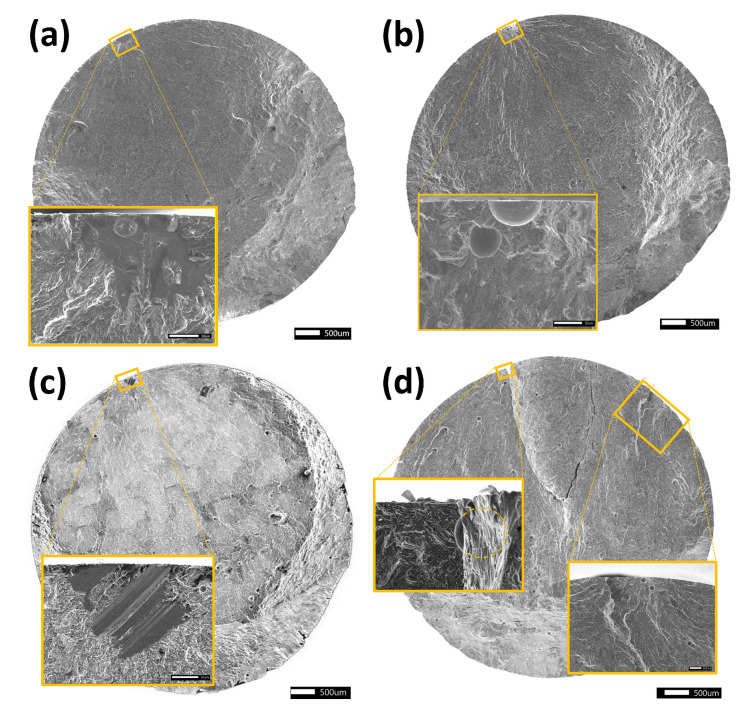
Examples of fracture initiating defects documented via SEM. (**a**) Specimen AL1 with initiation at an LOF defect. (**b**) Specimen AL2 with initiation at a pair of pores. (**c**) Specimen BL3 with multiple initiations on the fracture surface. (**d**) Specimen BL3 with multiple initiations. To the left, a large initiating pore barely visible behind an overhang. The approximate hidden shape is marked with a dashed line. To the right, initiation at a pair of pores.

Three fractures initiated from LOF defects, all from Build A. Two specimens with LOF initiation were tested at R=0 and 500 MPa (specimens AH4 and AL4) and the third at R=0 (AL1). For all other specimens, the fractures initiated from pores. Four specimens had multiple initiation sites visible on the fracture surface (AH3, BH1, BH3, and BL3). For these cases, the measurements of the “distance from surface” and “initiator radius” were at the initiation with the largest visible crack propagation area. All initiations started at the specimen surface, and examples of typical fracture surfaces are shown in Figure 6. The figure demonstrates initiations from different configurations of defects such as LOF, interacting pores, and singular pores. It also shows one fracture surface with multiple initiating defects.

### 3.2. X-ray Computed Tomography Data

**Figure 7:**
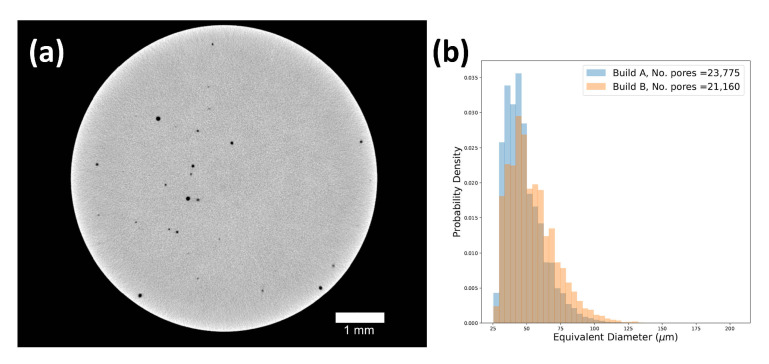
(**a**) Tomogram showing the porosity in a test specimen. (**b**) Distributions of the defect size (equivalent diameter) of segmented defects. Data are combined from XCT scans of six specimens from each of the two builds.

Defect analysis on the segmented XCT data showed between 2704 and 5214 spherical or near-spherical defects in each specimen. The spatial distribution of defects was seemingly random with both large and smaller porosity scattered in the volume. A large number of surface-adjacent defects of varying sizes were observed. The overall largest observed defect had an equivalent diameter of 209 μm. A typical tomogram, as well as the combined defect size distributions from the two EBM builds are presented in Figure 7. Build B (90 μm build layer height) showed slightly fewer defects in total, but a higher number of large defects than Build A (50 μm build layer height). The global level of internal porosity as evaluated by XCT is shown in Table 4.

Even though three of the sixteen fatigue specimen fractured from initiations at LOF defects, no such defect was visible in the XCT data. This is likely because of the flat geometry of LOF defects in combination with the limitations of the resolution. This was previously discussed in [20].

**Figure 8:**
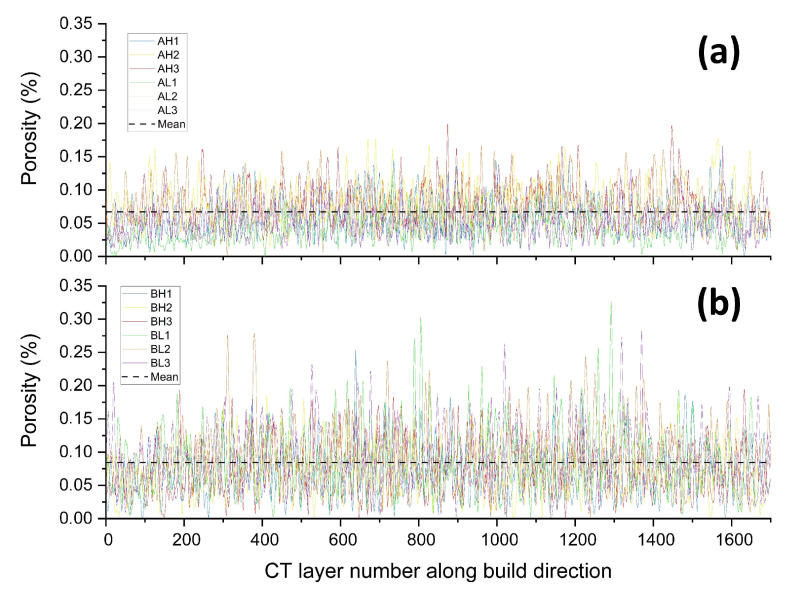
Porosity fraction along the building direction derived from XCT data for each build. Horizontal dashed lines show the global mean porosity for each build. (**a**) Build A. (**b**) Build B.

For the current method, it is important that the defect distribution is homogeneous in 3D. In a previous work, the defect distribution of the 12 XCT scanned specimens was shown to be homogeneous in the building plane (i.e., 2D) [20]. There, the authors proposed that the defect distribution was homogeneous in 3D since the scanned volume was machined from a region in the hatch, not adjacent to areas of production inhomogeneities such as the edges/contour interface or the top/bottom surfaces. In order to confirm homogeneity for further analysis in this study, the local porosity level of each tomogram along the building direction was calculated. The results are plotted in Figure 8. From the figure, it can be seen that there are three specimens that have horizontal planes (perpendicular to the building direction) with a porosity level higher than 0.25% (specimens BL1, BL2, and BL3), all from the lower part of Build B (90 μm build layer height). These planes all contain at least one of the 10 largest observed defects. Despite these outliers, the statement from [20] is considered valid. The average porosity levels across all scanned specimens as seen in Table 4 are marked with horizontal lines in Figure 8, with Build A showing a lower average porosity level than Build B.

### 3.3. Statistical Fitting

**Figure 9:**
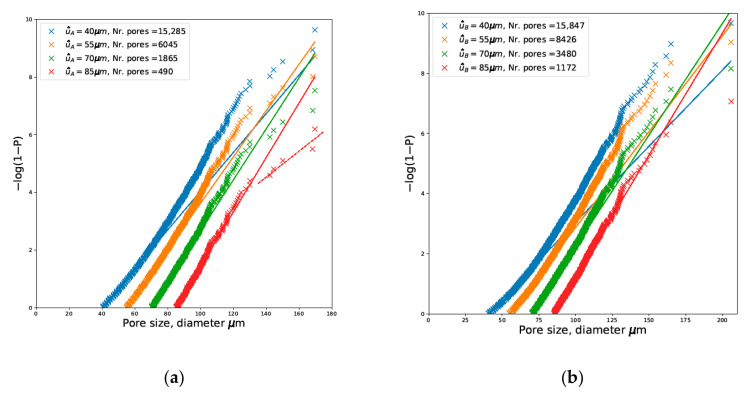
Linear fits to the probability plots of XCT data from Build A at various cut-offs. (**a**) Build A; the dashed line shows an example of a potential secondary linear fit to the extreme values of the distribution. (**b**) Build B.

Figure 9 shows the graphical evaluation of the cut-off and scale parameter through probability plot linear fits of the GPD to the defect size (diameter) distribution of the two builds. Various cut-offs are shown including the selected cut-off values. The resulting cut-off and scale parameter values, as well as the maximum defect sizes for 1000 specimens $\hat{x}{\left(T\right)}_{1000}$ are listed in Table 5.

### 3.4. Fatigue Life Prediction

**Figure 10:**
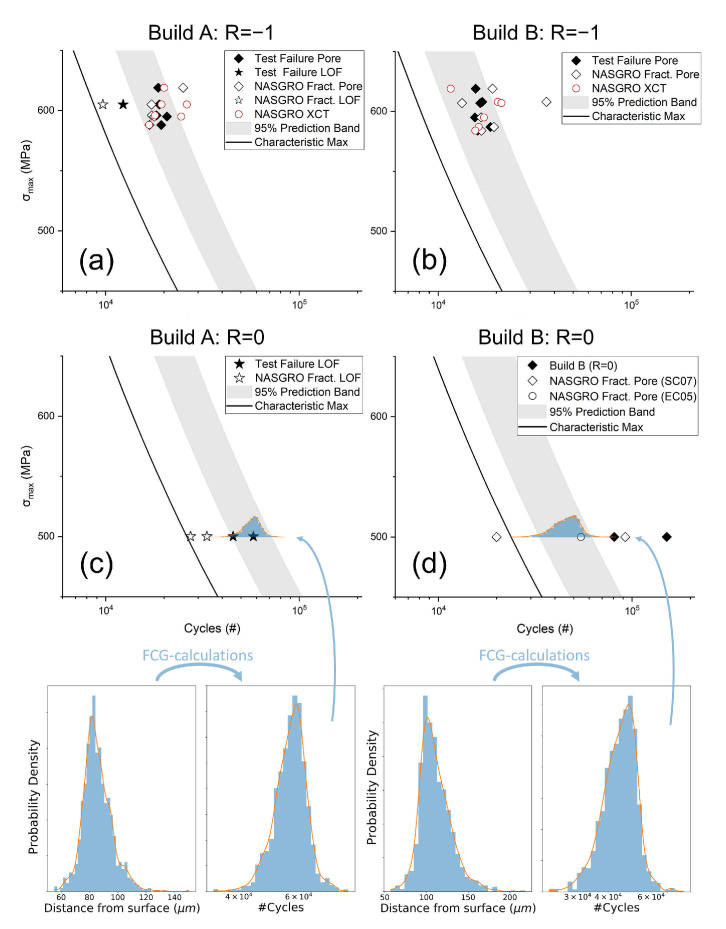
Experimental fatigue data (solid markers) and modelled life predictions (hollow markers and bands). (**a**) Build A, R=−1. (**b**) Build B, R=−1. (**c**) Build A, R=0. (**d**) Build B, R=0. Histograms show the distributions of the distance of simulated defects to the specimen surface and the distributions of calculated life. FCG, fatigue crack growth.

The result from the predictive modeling is presented in Figure 10. The figure shows experimental fatigue data (solid markers) and deterministic fatigue life predictions from fractography and XCT data (hollow markers). The correlation between experimental and predicted fatigue life is satisfying. Deterministic prediction from fractography is non-conservative for the specimens that failed from LOF defects. The figure also shows probabilistic fatigue life prediction bands, covering the 2.5–97.5 percentile range of modeled life. All experimental fatigue life data for specimens tested at R=−1 and failing at pores fall within the prediction bands. For specimens tested at R=0, the deterministic predictions are conservative, as are the probabilistic prediction bands. The solid lines in Figure 10 are lower bound estimates based on the characteristic maximum defect size for 1000 specimens $\hat{x}{\left(T\right)}_{1000}$. All experimental fatigue life data are to the right of these lines. The distributions of simulated defect distances to the surface and corresponding life distributions at $\sigma_{max}=500$ MPa and R=0 are shown at the bottom of Figure 10. As can be seen, the life distributions are skewed to longer lives. Miniature versions of the life distributions are visible in Figure 10c,d.

## 4. Discussion

### 4.1. Defect Distribution Simulations

The use of extreme value statistics and POT is feasible when working with XCT data due to the large amount of available data points. If a proper XCT setup is selected, the relative measurement error related to resolution primarily affects the detection of defects below the selected cut-off, which is another advantage of the POT sampling method. However, in order to further investigate the suitability of the POT sampling method, two important topics to discuss are the selection of the cut-off and if the material volume investigated by XCT is sufficiently large.

#### 4.1.1. Selecting a Cut-Off

For selecting the cut-off parameter in the two-parameter exponential distribution (2), there are multiple options. Completely objective methods such as using pre-selected cut-off values or distribution percentiles are the fastest ways, but are not always optimal. In the current work, a graphical approach was used, requiring more subjective analysis of the dataset in the selection. This could be preferable in many situations since, using an objective method, it is not unlikely that a parameter value can be rejected even if the deviation from the data is negligible from a practical point of view. This is especially true for large data sets due to the large influence of the data collection on the parameters [37].

Validation of the selected cut-off diameters can be done by comparing the selected cut-offs in Table 5 (70 μm for Build A and 85 μm for Build B) to the radii of the fracture initiating pores listed in Table 3. It was observed that two specimens showed the largest initiators that were marginally smaller than the selected cut-off diameter (specimen AH3 with a diameter of 68.8 μm and BL3 with diameter 79.4 μm). However, in both of these cases, the specimens showed more than one initiation site and did thus not fully represent the crack case used for modeling, which assumed failure from a single initiation. The other specimens all fractured from defects with sizes larger than the cut-off diameters. This validates the selected cut-off values since a higher value would have excluded defect sizes that in reality lead to failure from the defect distribution simulations. A lower cut-off value selection would have given more weight to the small defects in the maximum likelihood fit and would then poorly describe the largest defects, as illustrated in Figure 9, where the fit is poor for larger defects at smaller cut-off values.

In Figure 9a, some indication of two separate defect populations can be seen (solid and dashed lines) in Build A. Romano et al. [26] made similar observations when combining the size measurements of LOF defects and pores into a single distribution. They separated the two defect types based on sphericity criteria since it is not advised to assess the effect of different defect types with a single distribution [35]. It is unlikely that the largest (extreme) defects observed in this work are LOF defects mainly due to their overall shape, which is round and slightly flattened in the build layer plane and not flat with jagged edges like the observed LOF defects. This morphological observation led to the conclusion that all observed defects should be considered to be of the same defect type and thus be described by a single extreme value fit. The fact that these defects did not line up with the bulk of the porosity in the probability plots was attributed to the selected measure of defect size being the projected area in the build plane.

**Figure 11:**
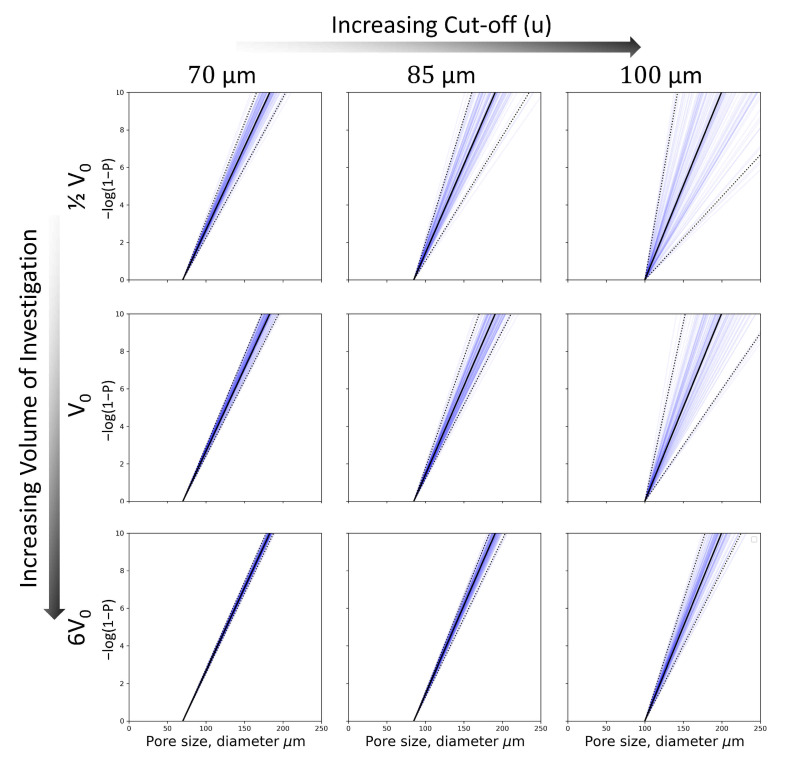
Scatter of the extreme value estimation of σ with increasing cut-off (*u*) and simulated volume. $V_{0}$ is the volume of one test specimen. The scatter is illustrated by maximum likelihood fitted lines to exponential probability plots of the simulated specimens. Each sub-plot contains 100 lines with the mean slope in solid black and the fifth and 95th slopes in dotted black. The bottom left plot represents the scatter of the estimation of σ in the current work.

One step of the defect distribution simulation, as described in Section 2.1, is the simulation of all defects of a size exceeding the cut-off diameter in a single specimen volume. By fitting the distribution scale parameters σ to a simulated sample population, it is possible to illustrate the effect of different cut offs (*u*) on the scatter of σ and, by extension, the sampled defect sizes. This is presented in Figure 11 for simulations of specimens from Build A. At increasing cut-off values, there is an associated increase in the scatter of the scale parameter due to the reduced number of simulated defects. By extension, this increases the scatter in the fatigue life estimation due to the reduced probability of simulating large defects at the specimen surface. There was little variation in the mean of the scale parameter from $\hat{\sigma }$, which is to be expected since the defect sizes for each specimen simulation themselves were sampled from a distribution with a fixed scale parameter.

#### 4.1.2. Effect of Investigated Volume

In addition to the cut-off selection, the total material volume investigated by XCT also plays a role in the scatter of the extreme value estimation of σ. A larger scanned volume equals more detected defects, informing the statistical parameter fitting. In the current work, six fatigue specimen gauge sections ($6V_{0}$) of each of the two builds were scanned by XCT. The effect of scanned volume is illustrated in Figure 11 by simulating six specimens to which the maximum likelihood fit was made. At the selected cut-off (70 μm), there was little scatter with the change in scanned volume. This shows that the amount of XCT used in this work adds negligibly to the uncertainty in the scale parameter estimation. On the other hand, the cut-off selection process is more sensitive to changing the statistical sampling uncertainty. In fact, scanning only one specimen rather than six adds about the same amount of parameter scatter as increasing the cut-off from 70 μm to 85 μm, an increase >20%. Due to the cost of XCT, it is worth pointing out that even the XCT of a rather limited volume, in this case one gauge length ($V_{0}\approx 475$ mm^3^), would likely have been sufficient for the extreme value statistical analysis of the material presented given the XCT resolution and data processing used in this work.

Ultimately, any scatter in the extreme value estimation translates to a decreased predictive accuracy i.e., a wider predictive scatter band. With six specimens scanned for each material and the cut-offs selected, prediction scatter bands became narrow but also remained accurate to the simulated data when compared in a post predictive check.

### 4.2. FCG Calculations

#### Crack Case

The choice to use the “semi-elliptical surface crack in a solid cylinder” case (SC07) in NASGRO limited the simulations to only consider cracks originating from porosity at or close to the surface. Considering the high likelihood for fatigue critical cracks to originate from the surface region [20,21,22], this seems like an acceptable, even preferred, limitation. This is further supported by the fracture surface observations in this study, which showed that the fracture in thirteen out of sixteen specimens originated from defects fulfilling the Murakami criterion (r/h>0.8) and two out of sixteen barely failing the criterion. The only exception was specimen BL4 with r/h=0.466. This specimen also had the largest initiating non-LOF defect. Since this defect was further from the surface than the other initiating defects, the NASGRO estimate from fractography measurements showed a conservative (>1) A/P (actual/predicted) life ratio value of 4.05 (Table 3). This is an effect of the selected crack case. Since the only input parameter in SC07, *a* (distance from the surface), increases with increased distance from the surface, the initial size (area) of a defect not immediately at the surface will be overestimated. This is most clearly seen in Figure 10d where the FCG calculation from the fractography measurement of specimen BL4 showed a shorter life than the already conservative characteristic maximum estimate. The fact that one specimen fractured from a defect not considered near the surface according to the Murakami criterion raises questions regarding the use of the Murakami criterion as the screening method for non-critical cracks if a surface crack case is used. An alternative to using the Murakami criterion could be to consider all defects within a fixed sub surface volume to be near the surface and thus included in the analysis. This would require determination of such a volume whilst accounting for the geometry of the defects present in the specific material, which can be challenging [42]. Another solution that avoids the need to classify defects as near the surface would be to consider a different (internal) crack case. This way, ready-made stress intensity factor solutions for internal cracks would allow for the life estimation of all simulated cracks in the current work. There are two main issues with this approach, the first being that it is inefficient. Since there is a clearly documented affinity for crack formation at or near the surface (not least under the tested conditions), a majority of crack calculations on internal defects would not be interesting for actual life prediction. The second issue is that it is practically difficult to find ready-made stress intensity factor solutions for anything but the simplest geometries. For example, NASGRO v9.10, a state-of-the-art fatigue crack growth software, does not yet include any stress intensity factor solution for internal cracks in cylindrical bodies. Looking at the available options for internal crack solutions in NASGRO, a comparison was made between surface and embedded crack cases, the result of which is included in Figure 10d. The embedded crack case used was “elliptical embedded crack (offset) in plate subject to uni-variant stress” (EC05). Keeping the cross-section area between the circular SC07 case and square EC05 equal, the A/P ratio decreased from 4.05 to 1.49. This indicates that a prediction based on surface crack cases is unsuitable for cracks that are near the surface, but not directly at the surface and that using a crack case for an embedded crack is a better alternative, though it is unclear how valid this claim is in a general case. In a defect-rich material, it is likely that there is a competition between defects in regards to which crack initiation leads to failure. Provided there are no large defects at the surface, this could mean that at lower loads, crack initiation from smaller defects that are at the surface are deactivated, leading to a transition from surface-originating failure to failure from sub-surface defects. This is an effect that has previously been observed in AM produced stainless steel alloy 316L containing large sub-surface defects [43]. If this transition exists in material containing smaller internal defects than reported in [43], the prediction model in this work could be overly conservative since it assumes critical cracks to originate at the specimen surface.

### 4.3. Fatigue Life Prediction

Figure 10 summarizes the results of the life predictions from crack growth calculations. There is a difference between the two test conditions, not only in the accuracy of prediction, but also in the spread of tested life. At Δϵ≈1% and R=−1, all twelve specimens fractured at around 20,000 cycles, whereas at $\sigma_{max}=500$ MPa and R=0, there was more scatter despite fewer specimens being tested.

#### 4.3.1. Deterministic Prediction from Fractography

Crack growth calculations based on the measurements of the positions of the actual initiating defects on the fracture surfaces yielded varied results. For specimen tested at R=−1 with pores as initiators, the A/P values as presented in Table 3 ranged from 0.465 to 1.27 with a majority remarkably close to one. The one outlier, the non-conservative 0.465 value, was likely due to the specimen fracturing from multiple initiations, meaning that the defect measured for prediction did not solely cause the fracture. For the R=0 tested specimens, two out of four fractured at pores, both in specimens from Build B. As previously discussed, the A/P ratio of 4.05 can be attributed to the overestimation of initial crack size due to using a surface crack case. The second A/P value of 1.64 was slightly higher than what was observed for higher loads and a lower R-ratio, which might be attributed to the Paris crack growth description being less representative of the true crack growth at lower stress intensity factors. For the three specimens breaking from LOF defects, the fractography-based predictions were also slightly conservative with A/P values of 1.27, 1.66, and 1.74. Because of the limited number of specimens fracturing from LOF defects, the results are inconclusive, but the results still build confidence in the current crack propagation approach as a tool for life prediction since they are relatively accurate and baseline conservative when using data from true initiating defects, which should be preferred over a non-conservative life estimate.

#### 4.3.2. Deterministic Prediction from XCT

Since fractography requires destructive mechanical testing, a non-destructive XCT-based prediction would be more useful for prediction. The most direct way of doing this would be to identify a worst case defect among the defects visible in the XCT data from each scanned specimen. This is done using the same method as when sampling from simulated specimens, i.e., by sorting the depth of defects fulfilling the Murakami criterion. Using these direct observations as the crack propagation input, the resulting lives are also plotted Figure 10. The results were similar to the predictions made from fracture surface measurements in that they were very close to the actual life with a mix of slightly conservative and non-conservative predictions. Specimen AL1, the only XCT scanned specimen that fractured from an LOF defect, was the least accurate prediction with an A/P of 0.469. This was expected since no defect identified as an LOF defect was observed. The fact that these results are as close or closer to the actual life than predictions from fractography is promising since XCT could be carried out on actual components intended for use. The main challenge of this approach is that it is inefficient in that almost none of the recorded defect information is used. For quality control, it would also require the entirety of the loaded volume of a part to be inspectable by XCT. This is likely to be neither practical, nor time efficient for a real production scenario.

#### 4.3.3. Probabilistic Prediction from XCT

In theory, a probabilistic prediction based on the statistical simulation of defects has the advantage of extrapolation, being scalable to work for parts of material that are not directly inspectable by XCT. For both Build A and Build B, the width and slope of the 95% prediction bands in Figure 10 are similar for the two R-ratios with a slight translation in life at equivalent $\sigma_{max}$ values. This is to be expected as, for each build, the same defect depth distribution was used as the input to all life calculations. The life distributions at R=0 and $\sigma_{max}=500$ MPa are shown in Figure 10. At higher loads (Figure 10a,b), the predictive bands entirely included the test data apart from one specimen, which fractured from an LOF defect. The same is not true at lower loads. Like the case with the direct predictions at lower loads, this could indicate a difference in the accuracy of the model in that exclusively considering defects fulfilling the Murakami criterion is too strict of a condition for accurate prediction. Nevertheless, the predictive bands were conservative and within a factor two of tested life, which shows the predictive capabilities of the method at the conditions studied.

The fatigue lives and predicted lives of all specimen, including those fracturing from an LOF defect, fell above the conservative life prediction. This is to be expected, but also confirms that the simple approach of evaluating a characteristic maximum defect ($\hat{x}\left(T\right)$) from an XCT size distribution and placing it in a critical location for failure is a feasible method for lower bound prediction.

## 5. Conclusions

This work used X-ray computed tomography and extreme value statistics combined with fracture mechanics to predict the fatigue life of electron beam melted Ti-6Al-4V test specimens. The following conclusions can be drawn:

The life predicted from deterministic fatigue crack growth calculations based on fractography was accurate, but showed mixed conservative and non-conservative predictions. This was found to be due to crack initiations not agreeing with model assumptions (single initiation from a defect at the surface). The life predicted from deterministic fatigue crack growth calculations based on “worst-case defects” detected by XCT were found to be accurate. One drawback of using XCT in this manner is the inefficient use of defect information. It was also observed that XCT can fail to detect fatigue-critical LOF defects due to resolution issues.

The proposed probabilistic predictive model was shown to be conservative and accurate for one of the tested conditions (R = −1, 1% strain). The model was conservative and satisfactorily accurate, within a factor of two, for the other tested condition (R = 0, 500 MPa). The predicted lower bounds of the fatigue life, estimated from the characteristic maximum defect size, were shown to be valid conservative estimates as no specimen, including those with LOF initiations, fractured outside these bounds.

## Figures and Tables

**Table 1:** Chemical composition of the as-delivered powder compared to ASTM requirements.

Element	C	O	N	H	Fe	Al	V	Y	Other	Ti
ASTM F3001	<0.08	<0.13	<0.05	<0.012	<0.25	5.50–6.50	3.50–4.50	<0.005	<0.40	Balance
As-delivered	0.01	0.09	0.02	0.002	0.18	6.35	4.01	<0.001	<0.40	Balance

**Table 2:** Settings used for XCT scans.

Field of View (mm)	Voxel Size (μm)	Tube Voltage (kV)	Tube Output (W)	# of Projections	Exposure Time (s)	Scan Time (h)
17.4	8.5	140	10	1601	9	5.5

**Table 3:** Results from the fatigue testing and prediction based on fractography measurements. If multiple crack initiation sites were found, only the largest initiator is listed. *r* and *h* are parameters in the Murakamis criterion (1). h=0 indicates that the center of the equivalent spherical initiator is outside the specimen surface. Stress values are midlife stresses. * *More than one initiation site contributed to failure*. ^†^ *Crack initiation at a lack of fusion (LOF) defect. A, Build A; B, Build B; H, higher; L, lower*.

Specimens	$\sigma_{\max}$ (MPa)	$\sigma_{\min}$ (MPa)	Initiator Radius (r) (μm)	Distance from Surface (h) (μm)	r/h	Actual Life (A) (# of Cycles)	Predicted Life (P) (# of Cycles)	A/P
**R = −1**							
AH1	596	−633	41.8	51.7	0.809	18,340	17,359	1.06
AH2	605	−616	38.6	51.4	0.751	18,975	17,295	1.10
AH3 *	619	−608	34.4	17.6	1.96	18,712	25,172	0.743
AL1 ^†^	605	−614	-	-	-	12,334	9690	1.27
AL2	595	−608	40.0	52.4	0.763	20,763	17,865	1.16
AL3	588	−633	50.6	50.7	0.998	19,396	16,881	1.15
BH1 *	608	−618	59.3	0	∞	16,935	36,395	0.465
BH2	587	−655	45.2	39.8	1.14	18,672	19,465	0.959
BH3 *	619	−614	49.9	23.2	2.15	15,642	19,145	0.817
BL1	595	−628	54.0	44.7	1.21	15,514	16,702	0.929
BL2	584	−653	58.3	44.9	1.30	16,022	16,778	0.955
BL3 *	607	−616	39.7	44.1	0.899	16,527	13,303	1.24
**R = 0**							
AH4 ^†^	500	0	-	-	-	45,497	27,455	1.66
AL4 ^†^	500	0	-	-	-	57,909	33,328	1.74
BH4	500	0	64.1	0	∞	151,075	92,230	1.64
BL4	500	0	78.5	169	0.466	80,959	19,985	4.05

**Table 4:** Average porosity levels as evaluated from XCT.

	Investigated Volume (mm^3^)	Defects (#)	Porosity (%)
**Build A**	2769.1	20,486	0.0672
**Build B**	2769.5	22,803	0.0844

**Table 5:** Cut-off ($\hat{u}$) and scale ($\hat{\sigma }$) parameters, as well as the characteristic maximum defect size for 1000 specimens $\hat{x}{\left(T\right)}_{1000}$.

	$\hat{u}$ (μm)	$\hat{\sigma }$	$\hat{x}{\left(T\right)}_{1000}$ (μm)
**Build A**	70	11.295	192.2
**Build B**	85	12.31	211.4

## Data Availability

The data presented in this study are available on request from the corresponding author. The data are not publicly available due to, in part, being proprietary to GKN Aerospace Sweden AB.
